# A Systematic Review of Radiotherapy Capacity in Low- and Middle-Income Countries

**DOI:** 10.3389/fonc.2014.00380

**Published:** 2015-01-22

**Authors:** Surbhi Grover, Melody J. Xu, Alyssa Yeager, Lori Rosman, Reinou S. Groen, Smita Chackungal, Danielle Rodin, Margaret Mangaali, Sommer Nurkic, Annemarie Fernandes, Lilie L. Lin, Gillian Thomas, Ana I. Tergas

**Affiliations:** ^1^Department of Radiation Oncology, Perelman School of Medicine, University of Pennsylvania, Philadelphia, PA, USA; ^2^Johns Hopkins School of Public Health, Baltimore, MD, USA; ^3^Department of Gynecology and Obstetrics, Johns Hopkins Hospital, Baltimore, MD, USA; ^4^Department of Surgery, University of Western Ontario, London, ON, Canada; ^5^Department of Radiation Oncology, University of Toronto, Toronto, ON, Canada; ^6^Department of Obstetrics and Gynecology, Columbia University College of Physicians and Surgeons, New York, NY, USA

**Keywords:** radiation capacity, global health, low- and middle-income countries, radiation oncology access, systematic review, systematic review

## Abstract

**Objectives:** The cancer burden in low- and middle-income countries (LMIC) is substantial. The purpose of this study was to identify and describe country and region-specific patterns of radiotherapy (RT) facilities in LMIC.

**Methods:** A systematic review of the literature was undertaken. A search strategy was developed to include articles on radiation capacity in LMIC from the following databases: PubMed, Embase, CINAHL Plus, Global Health, and the Latin American and Caribbean System on Health Sciences Information. Searches included all literature up to April 2013.

**Results:** A total of 49 articles were included in the review. Studies reviewed were divided into one of four regions: Africa, Asia, Eastern Europe, and South America. The African continent has the least amount of resources for RT. Furthermore, a wide disparity exists, as 60% of all machines on the continent are concentrated in Egypt and South Africa while 29 countries in Africa are still lacking any RT resource. A significant heterogeneity also exists across Southeast Asia despite a threefold increase in megavoltage teletherapy machines from 1976 to 1999, which corresponds with a rise in economic status. In LMIC of the Americas, only Uruguay met the International Atomic Energy Agency recommendations of 4 MV/million population, whereas Bolivia and Venezuela had the most radiation oncologists (>1 per 1000 new cancer cases). The main concern with the review of RT resources in Eastern Europe was the lack of data.

**Conclusion:** There is a dearth of publications on RT therapy infrastructure in LMIC. However, based on limited published data, availability of RT resources reflects the countries’ economic status. The challenges to delivering radiation in the discussed regions are multidimensional and include lack of physical resources, lack of human personnel, and lack of data. Furthermore, access to existing RT and affordability of care remains a large problem.

## Introduction

As populations’ age and infectious disease control extends lifespan, cancer and other non-communicable diseases are becoming increasingly significant burdens of mortality in low- and middle-income countries (LMIC) (1). Over 70% of cancer cases will be diagnosed in LMIC by 2030 (2). Yet most developing countries do not have the resources or infrastructure to prevent, diagnose, or treat this growing burden of cancer (2). Compounding the issue is the lack of cancer registries and cancer treatment capacity in most of the developing world. Existing data represents only a fraction of the true burden of cancer, with our best estimates being estimates at best.

Leading medical and public health organizations have spearheaded international initiatives to increase awareness of this issue, but great needs still exist (3). One organization, the International Atomic Energy Agency (IAEA), has organized the Directory of Radiotherapy Centres (DIRAC), which acts as a central record and quantification of international radiotherapy (RT) capacity. Apart from DIRAC, few reports exist that describe the capacity requirements necessary to deliver RT. This capacity includes country-specific infrastructure, equipment, personnel training, quality assurance, and challenges surrounding RT facilities. The objective of this study was to perform a systematic review of RT capacity in LMIC as documented in the literature. In addition, we aimed to compare reports in the literature to that of reports from the IAEA.

## Materials and Methods

We searched PubMed (1946 to April 2013), Embase (1974 to April 2013), CINAHL Plus (1937 to April 2013), Global Health (1910 to April 2013), and the Latin American and Caribbean System on Health Sciences Information (LILACS) (1982 to April 2013). A core strategy was developed in PubMed and then translated for each database. All search strategies were developed using a combination of controlled vocabulary and keyword terms to define the concepts of radiation therapy, health services, and LMIC. Searches were run on April 19, 2013. (See Supplementary Material for more details on search strategies.)

All citations were imported into a reference management system and duplicates were removed. All citations were reviewed by two authors at the title and abstract level for pre-defined inclusion and exclusion criteria as defined below. A third author resolved disagreements between the initial two reviewing authors.

Articles on radiation capacity and facilities in LMIC were included. Articles not including low- or middle-income countries (as determined by the World Bank, see Supplementary Material for complete list) and radiation facilities or capacity were excluded.

**Figure 1:**
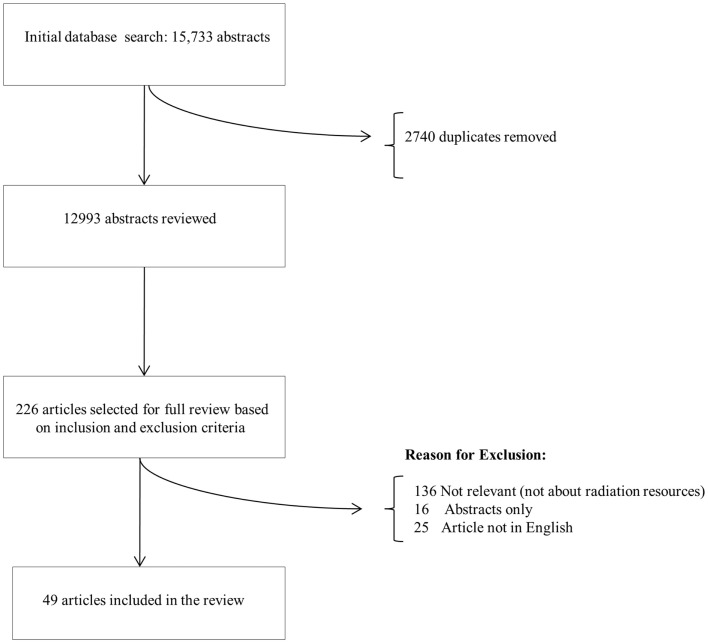
**Article selection**.

Based on the initial database search, abstracts were selected for final review (Figure 1). If they met the above inclusion criteria, they were selected to be included in the review.

## Results

All the studies included in the review were divided into one of the four regions: Africa, Asia, Eastern Europe, and South America. Each of the four regions will be described separately.

### Africa

#### Countries covered

A total of 16 articles covering the Africa region were included in this review in Ref. (4–19). The countries covered were: Ghana, Liberia, Nigeria, Sierra Leone, South Africa, and Uganda (4, 7–10, 13, 14, 16, 19). Publication dates ranged from 1972 to 2013 (7, 11, 19). Six articles provided reviews and surveys on the continent as a whole and one article reported on developing countries in general (5, 6, 11, 12, 15, 17, 18). Radiation capacity is not discussed for a majority of countries on the African continent. The two most recent articles present updated data for the African continent: Denny and Anorlu reviewed cervical cancer in Africa and the IAEA reported on the status of RT resources in Africa (5, 11).

#### Cancers treated

The most common cancer addressed was cervical cancer, though seven articles included data on non-gynecological cancers (8, 11, 12, 14, 15, 17, 18). Advanced cervical cancer is treated with radiation, a combination of external beam radiation and brachytherapy. It is estimated that 80,000 African women are diagnosed with cervical cancer each year and approximately 60,000 die of the disease annually, though validation of these estimations is difficult due to limited availability of cancer registries (6). RT is frequently the first line of treatment for cervical cancer, and, in a single institution survey, up to 97.3% of newly diagnosed patients were referred for RT (13). However, the article did not describe where these centers are located and how many women were actually treated or able to access these centers. Unfortunately, many women do not present for follow-up at these tertiary care centers, which makes it challenging to evaluate outcomes. Radiation is also used for palliative treatment with notable improvement in survival (12). However, the 5-year cervical cancer survival rate continues to be low, ranging from 15 to 30% in Africa compared to 60% in North America (12).

#### Available equipment

According to the latest update from the DIRAC database, there are currently 160 RT centers on the African continent (11). A total of 88 cobalt-60 machines, half of which are over 20 years old, and 189 linear accelerators are operating in those 160 centers. Sixty percent of machines are concentrated in Egypt and South Africa, while 29 of 54 countries in Africa are still lacking any RT resource. Given the ideal ratio of 4–8.1 RT centers per 1 million people or 1 MV per 250,000 people, as defined by the IAEA, every country in Africa needs more centers and machines (17). The highest capacity is in Mauritius with 2.36 centers/1 million people followed by South Africa with 1.89, Tunisia with 1.55, and Egypt with 0.93 (17). Not surprisingly, there appears to be a correlation between Gross National Income (GNI) and RT capacity (11).

There are limited reports from most of West Africa with the exception of Nigeria. Several reports on Nigeria from as early as 1972 record a gradual increase in RT delivery capacity over time. In 1972, the longest standing RT center had been in existence for 20 years, housing one linear accelerator and two sets of brachytherapy applicators (7). Between 1972 and 1990, reports from West Africa suggested that there were a total of two RT centers that served Ghana, Liberia, Nigeria, and Sierra Leone (8). During this time, cervical cancer patients were treated with brachytherapy alone. In 2000, the University College Hospital in Ibadan, Nigeria, reported a 500 case retrospective review where combined external beam RT and low dose rate brachytherapy was used to treat patients with cervical cancer, an improvement from previous reports where hospital resources allowed for only monotherapy with low dose rate brachytherapy (4, 7). In 2008, five RT centers were in operation in Nigeria, with more expected to come (10). Despite the gradual increase in RT centers, waiting lines for these machines continue to be long. Nigeria and the surrounding West African countries are clearly operating under capacity.

#### Human resources

Data on available RT human resources were limited with specific numbers only available for South Africa. In 1994, with a population of 24 million, South Africa had 58 practicing radiation oncologists, 190 therapy radiographers, and 30 medical physicists, which represented only a fraction of total registered professionals (14). With 58 radiation oncologists, South Africa had only 1 radiation oncologist per 350 patients, falling short of the recommended IAEA ratio of 1 radiation oncologist per 200–250 patients (20). In 2011, a review of cervical cancer treatment in Africa reported that “training facilities in cancer diagnosis and management” were few and only found in Algeria, Egypt, Libya, Morocco, Nigeria, South Africa, and Zimbabwe (6). It was unclear whether the curriculum covered RT or if the types of health care professionals trained (physicians vs. nurses vs. technicians) would be able to deliver RT after completion of the program. Multiple articles also emphasized the critical lack of pathology and laboratory services needed to make the initial diagnosis of gynecological and other cancers (12, 15). The articles from Nigeria provide limited records of their human resources. One mention is made of the RT center in 1972, where only one Cambridge-trained medical physicist was noted to be available to the entire hospital (7).

### Americas

#### Countries covered

A total of five full articles covering Central America, South America, and the Caribbean were included in this review in Ref. (21–25). The countries covered were: Argentina, Bolivia, Brazil, Chile, Colombia, Costa Rica, Cuba, Dominican Republic, Ecuador, El Salvador, Guatemala, Haiti, Mexico, Nicaragua, Panama, Paraguay, Peru, Uruguay, and Venezuela. Four articles provided surveys of multiple South American countries, one article reported on Mexico alone, and one abstract reported on Brazil alone. No data were found for 20 of 26 countries in this region, including Belize, Grenada, Guyana, Honduras, Jamaica, and Suriname. The earliest article was published in 1984 and detailed RT resources throughout nine Latin American countries. The next most recent survey of Latin America was published in 2004 by the IAEA, and included 19 countries that account for 96% of the cancer cases in South and Central America and the Caribbean. The most recent article included in this review was published in 2013 and reported RT patterns in Mexico. Data from this region are sparse and available for limited time periods.

#### Cancers treated

The data on the most common cancers treated in this region were limited. In 1984, the report from nine Latin American countries found the most common cancer treated by radiation therapy to be cervical, followed by breast, head and neck, lung, and skin cancers (21). A 2013 report from Mexico suggested that the most common cancer treated by radiation therapy is breast cancer, followed by prostate, cervical, and lung (22).

#### Available equipment

The number of radiation machines in Latin America has increased over the past 30 years, especially in countries with greater populations. From 1989 to 2004, the number of machines in Brazil rose from 165 to 270 (64% increase) and, in Venezuela, from 18 to 44 (144% increase) (21, 23). The most recent information from 2004 lists the number of RT centers, cobalt-60 machines, and linear accelerators (linacs) from 19 Latin American countries (23). The total number of centers in the region was 470, with 710 machines, slightly more than half of which were cobalt-60 units (396 cobalt-60 units, 314 linacs). However, the distribution of these centers varied widely from country to country, ranging from 0 to 151. Although there has been a steady increase in the number of machines, the capacity remains insufficient, with an estimated 100 more teletherapy machines required to meet the IAEA guidelines of 1 machine per 500 new cancer cases per year (23). The quality of the machines and downtime was not discussed in more recent papers. In 1990, the majority of linacs in these countries were older machines operating at 4–10 MV without electron capability (24). Though the numbers of teletherapy machines in these countries are on the rise, it is unclear whether these have reliable power sources (for linear accelerators) and access to adequate servicing. Similar to Africa, there continues to be an insufficient number of machines to serve the populations in these countries.

Brachytherapy was offered at the majority of centers in Latin America, but the numbers of centers varied throughout the region. In 1990, all centers in Peru and Chile had brachytherapy for treatment of gynecologic malignancies, and 90% of centers in Brazil offered manual afterloading (24). Of 12 countries that provided data on brachytherapy, there were over 260 sets of cesium and radium manual afterloading devices, 23 cesium low dose rate afterloading devices, and 6 cesium high dose rate afterloading devices. However, the break down by country was not provided. In addition, there were 103 centers with iridium high dose rate units, 61 of which were in Brazil (23).

#### Human resources

In addition to an insufficient number of radiation therapy centers, there continues to be inadequate numbers of personnel trained to provide treatment. In 1983, of 27 radiation therapy centers studied in nine Latin American countries, 35.5% had an insufficient number of full-time radiation oncologists (<1 per 200–250 patients), 52% had an insufficient number of full-time physicists (<1 per 400 new patients), and only 15 of the centers had a dosimetrist available (21). However, they found that 25 of the 27 centers had an adequate number of radiation technicians (21). In 2004, the 19 countries studied had 933 radiation oncologists, with 642 more needed, representing a needed increase of 69% in number of radiation oncologists to meet IAEA standards (23). There were 357 medical physicists with 627 new physicists needed representing a 146% needed increase. There were 2300 radiation technologists, with 2500 more technologists needed (23). At the time of publication, only Bolivia and Venezuela had >1 radiation oncologist per 1000 cancer cases (23).

Formal training programs for radiation oncology are on the rise. In 1989, 10 out of 27 centers surveyed had radiation oncology residency programs and 14 offered formal training for radiation therapy technologists (21). By 2004, 12 of the 18 countries surveyed had postgraduate radiation oncology training at a total of 35 institutions, with the highest density of training in Argentina, Brazil, and Cuba (23). As of 2013, there are six centers in Mexico that were training radiation oncologists (22). Formal training of medical physicists is available in 7 of 18 Latin American countries at 22 centers (23). In Mexico specifically, two public universities offer a Masters in Medical Physics (22). However, the quality of these institutions and training programs was not described.

### Asia

#### Countries covered

A total of 20 full articles covering the Asia region were included in this review in Ref. (26–45). Of the countries, the United Nations Statistics Division classified as belonging to Asia, this systematic review covers the following LMIC: Azerbeijan, Bangladesh, Cambodia, China, India, Indonesia, Malaysia, Myanmar, Philippines, Saudi Arabia, Sri Lanka, Thailand, Turkey, and Vietnam. Nepal and Papau New Guinea were not included in the UN classification, but were included in the Asia region for the purposes of this review in Ref. (46). No data were found on Armenia, Bhutan, Georgia, Islamic Republic of Iran, Jordan, Kazakhstan, Kyrzygstan, People’s Democratic Republic of Lao, Tajikstan, Uzbekistan, or Yemen. The earliest article was written about Bangladesh in 1981 and the most recent article was written about India in 2013 (26, 45).

#### Cancers treated

Data on RT utilization were largely focused on treatment of cervical cancer (35–42). In the Philippines, 75.6% of new cervical cancer patients seen at Philippine General Hospital in 2008 were reportedly eligible for chemoradiation, yet financial constraints resulted in only 17.6% completing the recommended treatment course (39). In Indonesia, a total of 10,274 patients received RT in 2007. Eight centers were actively performing brachytherapy. In Indonesia, intracavitary insertions for cervical cancer represented the most common brachytherapy procedure (33). In Cambodia, a 2012 report noted 60 patients per day were treated with RT, but did not describe the distribution of cancer sites treated (31).

#### Available equipment

The most recent published survey of RT machines across Asia and the Pacific region was from the IAEA in 2001 (30). They report the number of RT centers ranged from 1 to 453, cobalt-60 machines ranged from 2 to 381, and linear accelerators ranged from 0 to 286 in countries in Asia. The number of cobalt-60 units far outweighed the number of accelerators, with the exception of Thailand and Malaysia, where the ratio of accelerators to cobalt-60 units was 1.08 and 2.71, respectively.

Some of the articles included in this review published after the 2001 IAEA report provide more updated figures on machine availability. Eav et al. reported that the RT department in Phnom Penh, Cambodia, was refurbished in 2003 with a second-hand cobalt-60 unit, x-ray simulator, and 2D dosimetry system, as well as a new remote afterloading brachytherapy machine (31). Two years earlier, when IAEA report was published, Cambodia did not have any reported equipment, reflecting the relatively rapid rate of change in the state of RT in Asia over the last decade. The plan for the new national cancer center in Cambodia includes two new linear accelerators and a high dose rate brachytherapy system.

In Turkey, as of 2011, there were 40 cobalt-60 units, 146 linear accelerators (1.8 linear accelerators per 1 million population), and 35 brachytherapy units (32). Large regional gaps were reported, however, with nearly 40% of linacs concentrated in two cities. As of 2008 in Indonesia, there were 22 RT centers, 17 cobalt-60 units, and 18 linear accelerators, which represent a very large increase over the 7 years since the 2001 IAEA report (33). This rapid equipment scale-up was also seen in India, where 12 additional linear accelerators were added over a 4-year period from 2001 to 2005 (34). In 2005, there were also 113 operational brachytherapy facilities, of which 44 were high dose rate units.

#### Human resources

Training of skilled personnel for RT was frequently cited as a major barrier to scaling up treatment delivery, despite a reported increase in human resource availability. In Cambodia, international partnerships between the University of Phnom Penh and other international centers, including Strasbourg University in France, has facilitated oncologist training (31). A 2-year program for general practitioners to obtain additional training in oncology has also been created, and, since 2011, a 5-year oncology specialization has been launched. There is currently one full professor oncologist in the country. In Indonesia, there was a 31% increase in RT personnel from 2004 to 2008 and the country has undertaken an expansion of its residency program (33).

In Turkey, the number of radiation oncologists has risen from 85 in 1985 to 446 by 2011, with an average of 30 new radiation oncologists entering practice per year (32). With this trend, Turkey will be in line with international benchmarks by 2023. There remains, however, a gap of 187–280 medical radiation physicists (representing a 10–65% personnel increase) and 600–800 RT technicians (representing 100–133% increase) in Turkey (32). To address these personnel gaps, additional university programs have been opened and working hours of existing staff have been extended.

### Eastern Europe

#### Countries covered

A total of two full articles covering the Eastern Europe region were included in this review. According to the United Nation Statistics Division and World Bank, the following countries are classified as LMIC in Eastern Europe: Belarus, Bulgaria, Hungary, Republic of Moldova, Romania, and Ukraine (47). The articles included covered the following four countries: Bulgaria, Hungary, Moldova, and Romania. No data were found on Belarus or the Ukraine. Both articles were international reviews; the updated results from the Patterns of Care for Brachytherapy in Europe (PCBE) was written in 2010 and the analysis of the European DIRAC database was written in 2013 (48, 49).

#### Cancers treated

The only available data on the most commonly treated cancers were found pertaining to brachytherapy in the PCBE study (48). Eastern European LMIC included in the analysis was classified in group II (Hungary) or group III (Bulgaria, Moldova, and Romania). In both group II and III, endometrial carcinoma was the most common cancer treated using brachytherapy (38% of cases in group II and 22% of cases in group III), followed by cervical cancer (31% of cases in group II and 57% of cases in group III). Both group II and group III treated more gynecological cancers with brachytherapy than group I, which consisted of high resource countries such as the United Kingdom, Germany, and France. This was attributed to higher incidence rates of uterine and cervical cancer in group II and III countries compared to group I.

#### Available equipment

The most updated information on the numbers of RT centers, cobalt-60 units, and linear accelerator machines were derived from the DIRAC database as follows: Bulgaria (13 centers, 10 cobalt-60 units, 5 linacs), Hungary (13 centers, 11 cobalt-60 units, 27 linacs), and Romania (19 centers, 16 cobalt, 12 linacs) (49). In Eastern Europe, cobalt-60 machines represent the majority of teletherapy machines, with linear accelerators accounting for only 31% of all teletherapy machines in countries like Bulgaria, Hungary, and Romania. The number of MV teletherapy machines per million people ranged from 1.3 in Romania to 3.8 in Hungary.

#### Human resources

No data were available on radiation oncology healthcare provider training programs.

### Developing world

Seven articles non-specific to a particular world region presented analyses of the cancer burden, resources, and demographic and economic trends affecting disease control in the developing world (50–56). Increased GNI and population size were found to be critical factors in the availability of radiation resources, with higher rates of equipment acquisition and an increased density of RT services in large and high-income countries (50). Experts observe that while knowledge, technology, and infrastructure to transport the technology are available, the lack of funding prevents scientific societies and international organization from transferring these resources to countries in need (51). The literature suggests that for developing countries, any plan to improve access to RT would need to be dynamic and multi-faceted, requiring buy-in at the levels of the local and state government, investment in staff training that is consistent across countries, increased physical capital and infrastructure, and improvement in patient cancer education programs (52, 53).

### Gaps in radiation facilities and gaps in published literature

To further characterize gaps in radiation facilities, we constructed a table comparing country-specific needs in radiation oncology infrastructure and the current state of available resources per the DIRAC database (Table 1) (57, 58). As seen in Table 1, except for a few LMIC, most countries are significantly lacking in their radiation infrastructure.

**Table 1:** **Comparison of estimated radiotherapy machines needed taking into account cancer incidence rates vs. the reported machine counts in the DIRAC database**.

Countries	# Annual cancer incidence	# Linacs + Cobalts needed	# Linacs + Cobalts (DIRAC)	# Brachy units needed	#Brachy units (DIRAC)
**AFRICA**					
Ghana	16580	4	1	2	3
Liberia	2148	2	0	1	0
Nigeria	101797	16	8	8	6
Sierra Leone	–	–	0	–	0
South Africa	74688	48	68	25	25
Uganda	27116	2	0	1	1
**AMERICAS**					
Argentina	104859	15	80	8	34
Bolivia	8689	3	1	2	5
Brazil	320955	33	285	17	135
Chile	36047	21	41	11	19
Colombia	58534	16	43	8	23
Costa Rica	7653	3	6	2	2
Cuba	31503	9	4	5	6
Dominican Republic	13063	5	10	3	2
Ecuador	20167	7	11	4	7
El Salvador	7782	5	3	3	2
Guatemala	14155	6	7	3	5
Haiti	8414	1	0	1	0
Mexico	127604	14	74	8	60
Nicaragua	5591	3	0	2	0
Panama	4630	5	5	3	1
Paraguay	7957	6	4	3	1
Peru	–	–	26	–	4
Uruguay	14584	7	14	4	6
Venezuela	36961	4	51	2	23
**ASIA**					
Azerbeijan	13123	2	4	1	1
Bangladesh	141086	10	9	6	3
Cambodia	–	–	0	–	1
China	2817210	87	1014	44	12
India	948858	19	187	10	305
Indonesia	292629	17	21	9	11
Malaysia	31998	13	37	7	5
Myanmar	–	–	1	–	5
Nepal	27768	9	3	5	2
Papau New Guinea	–	–	0	–	1
Philippines	77184	5	29	3	13
Saudi Arabia	–	–	29	–	9
Sri Lanka	24447	8	2	4	3
Thailand	112666	54	48	32	24
Turkey	95069	30	144	15	33
Vietnam	–	–	18	–	7
**EUROPE**					
Belarus	31188	9	11	5	12
Bulgaria	30701	8	5	4	15
Hungary	–	–	32	–	11
Republic of Moldova	9395	3	1	2	2
Romania	70262	8	19	4	6
Ukraine	142960	15	21	8	50

*Only countries covered in the systematic review are included. Number of annual incidence cancers is for all cancers, based on Globocan 2008 and NCI Radiation Research Program. Estimated number of linacs, cobalt-60 units (cobalts), and brachytherapy units needed were derived from the NCI Radiation Research Program and were based on the numbers needed to treat the one to two most populous cities in each country (58). Data from DIRAC was reported according to the most updated web database (57). DIRAC, Directory of Radiotherapy Centres. “–” symbol indicates no information available*.

Most of the recent literature in this review was derived from international databases; few articles were generated from within individual countries or regions reporting original, institution-specific, and up-to-date numbers. To highlight gaps in published literature, we compared the non-DIRAC-derived systematic review literature to the most recent DIRAC country-specific statistics (Table 2) (57). Only 11 countries out of the 47 included in this review had non-DIRAC-related publications. We extracted facility and equipment numbers based on non-DIRAC sources and found that most estimates were outdated and only Indonesia, Mexico, and Turkey had recent publications reflecting their current RT capacity. This demonstrates significant gap in published literature focusing on state of radiation oncology facilities in LMIC.

**Table 2:** **Comparison of radiotherapy resources described in reported literature vs. the DIRAC database (57)**.

Countries	# RT centers (literature)	# RT centers (DIRAC)	# Linacs + Cobalts needed	# Linacs (literature)	# Linacs (DIRAC)	# Cobalt-60s (literature)	# Cobalt-60s (DIRAC)	# Brachy units needed	# Brachy units (literature)	# Brachy units (DIRAC)
**AFRICA**										
Ghana (8)	0	3	4	–	1	–	3	2	–	3
Liberia (8)	0	0	2	0	0	0	0	1	0	0
Nigeria (5, 6)	5	9	16	–	8	–	5	8	–	6
Sierra Leone (8)	0	0	–	0	0	0	0	–	0	0
South Africa (14)	>13	39	48	20	68	19	11	25	>5	25
Uganda	–	1	2	–	0	–	1	1	–	1
**AMERICAS**										
Argentina (24)	–	82	15	12	80	80	36	8	–	34
Bolivia	–	5	3	–	1	–	5	2	–	5
Brazil (24)	–	222	33	55	285	110	63	17	–	135
Chile (24)	–	27	21	6	41	14	12	11	–	19
Colombia	–	55	16	–	43	–	35	8	–	23
Costa Rica	–	3	3	–	6	–	3	2	–	2
Cuba	–	9	9	–	4	–	10	5	–	6
Dominican Republic	–	9	5	–	10	–	3	3	–	2
Ecuador	–	10	7	–	11	–	6	4	–	7
El Salvador	–	4	5	–	3	–	3	3	–	2
Guatemala	–	8	6	–	7	–	3	3	–	5
Haiti	–	0	1	–	0	–	0	1	–	0
Mexico (22)	83	91	14	–	74	–	61	8	–	60
Nicaragua	–	1	3	–	0	–	2	2	–	0
Panama	–	2	5	–	5	–	0	3	–	1
Paraguay	–	3	6	–	4	–	1	3	–	1
Peru (24)	–	18	–	1	26	11	9	–	–	4
Uruguay	–	10	7	–	14	–	8	4	–	6
Venezuela	–	60	4	–	51	–	31	2	–	23
**ASIA**										
Azerbeijan	–	2	2	–	4	–	2	1	–	1
Bangladesh	–	14	10	–	9	1	12	6	1	3
Cambodia (31)	1	1	–	0	0	1	1	–	–	1
China (28)	–	1050	87	62	1014	186	516	44	–	12
India (27, 34)	129	314	19	47	187	184	333	10	113	305
Indonesia (33)	22	23	17	18	21	17	19	9	–	11
Malaysia	–	25	13	–	37	–	6	7	–	5
Myanmar	–	4	–	–	1	–	7	–	–	5
Nepal (35)	–	5	9	–	3	–	3	5	1	2
Papau New Guinea	–	1	–	–	0	–	2	–	–	1
Philippines	–	34	5	–	29	–	10	3	–	13
Saudi Arabia	–	12	–	–	29	–	1	–	–	9
Sri Lanka	–	7	8	–	2	–	11	4	–	3
Thailand	–	29	54	–	48	–	28	32	–	24
Turkey (32)	90	95	30	146	144	40	59	15	35	33
Vietnam (43)	9	19	–	3	18	13	19	–	12	7
**EUROPE**										
Belarus	–	12	9	–	11	–	21	5	–	12
Bulgaria	–	13	8	–	5	–	10	4	–	15
Hungary	–	13	–	–	32	–	11	–	–	11
Republic of Moldova	–	1	3	–	1	–	3	2	–	2
Romania	–	22	8	–	19	–	15	4	–	6
Ukraine	–	56	15	–	21	–	89	8	–	50

*Only countries covered in the systematic review are included. Data inferred from the literature contained only non-DIRAC or IAEA sources. Data from DIRAC were reported according to the most updated web database. DIRAC, Directory of Radiotherapy Centres. Cobalts, cobalt-60 units. “–” symbol indicates no information available*.

## Discussion

In this report, we present the results of a comprehensive systematic review of the literature on RT capacity in LMIC. Compared to IAEA recommendations, our review found an overwhelming lack of radiation oncology capacity relative to the large cancer burden faced by these populations (20). While the situation varies across regions and countries, many major challenges were similar. The most significant challenges reported include the quality and quantity of physical resources, the scarceness of human resources, and the unequal distribution of available resources. A recently published IAEA/DIRAC report reemphasized several of these issues (59).

Across regions, the number, age, and quality of machines contribute to suboptimal RT capacity. Many countries rely on machines that are more than 20 years old, which brings their functionality and reliability to question (11, 17). Because RT is first-line treatment for the vast majority of cervical cancers, many women with cervical cancer simply do not receive any treatment at all given the paucity of available RT centers. For example, in the Philippines, less than 20% of eligible women successfully receive radiation for their cancer (40). This is reflected in abysmally low 5-year survival rates for cervical cancer (15–30% in Africa) compared to higher income countries (60% in North America) (5, 6). While the numbers of centers providing radiation therapy in Latin American countries may be on the rise, the majority of these centers do not have simulation (81%) or treatment planning systems (55%) (23). The high upfront investment required at the local, state, and national government levels makes improving the quality and quantity of physical resources particularly challenging.

The lack of adequate human resources is another factor contributing to poor RT capacity in developing countries. Most reports on radiation oncology personnel availability and training indicate that there are not enough physicians and staff to treat the numbers of patients requiring radiation treatment. High patient volumes and lack of trained personnel often lead to long waiting lines and continued disease progression long after diagnosis. In Africa, there was only one report on radiation oncology personnel, which was specific to South Africa and reported that there were not enough radiation oncologists to meet the population’s needs (14). Although there are no published articles regarding human resources in other African countries, the situation is most likely similar, or more serious, than that of South Africa. In the Americas, the most recent survey of the region’s capacity reported the major constraint to adequate provision of radiation therapy was an insufficient number of specialists, rather than a lack of equipment (23). The inadequate number of personnel is in part due to an insufficient number of training programs for radiation oncologists, medical physicists, and radiation technologists. However, there has been a shift from a majority of radiation oncologists receiving training abroad to training locally; it is unclear what the impact of this will be on the numbers of providers of RT in these countries in the future (23). It is imperative that the availability of training in radiation oncology be improved to appropriately utilize existing physical resources, meet the maximum utilization potential, and account for attrition of workers over time.

The concentrated distribution of available radiation machines compounds the issue of limited capacity in many LMICs by restricting access to needed treatment. Generally, countries with higher GNI house the majority of radiation machines, with LMIC falling far short of the IAEA recommendations. Many countries do not have any radiation centers at all. For example, in Latin America, 75% of radiation oncology departments are located in the four most populous countries: Brazil, Mexico, Columbia, and Argentina (23). No published evidence suggests that Haiti has any regional access to radiation machines; however, neighboring Dominican Republic has three centers (23). It appears as if developed areas have a few large, high capacity centers, with the rest of the population having limited access to, at best, small, suboptimal centers (21). There is usually no mechanism in place for improving access for more rural populations and affordability of care remains a critical barrier (44).

Other culture, infrastructure, and systems issues contribute to poor capacity as well. In Africa, limited public knowledge and belief in traditional African healing contribute to more advanced disease at presentation, increasing requirements for palliative radiation and effective pain medication (4). While radiation can be very effective as palliative therapy, public information campaigns should go hand in hand with cancer prevention programs to urge women to seek medication attention earlier for better treatment outcomes (19). Another frequently highlighted issue was the difficulty in conducting a needs assessment for RT due to the lack of an organized cancer registry in many countries. Of the two articles about Eastern Europe, both were based on international registry and survey data. None originated internally within each country and therefore the RT capacity of this region remains limited to what is reported by DIRAC and IAEA.

Despite existing challenges, we discovered several countries working to improve their RT delivery systems. There are reports demonstrating slow and gradual increases in the number of RT centers in countries of West Africa (8). The number of Nigerian radiation centers and machines has been on the rise for over 30 years and is now also serving other countries of West Africa (7, 8, 10). Recently, there have been substantial increases in teletherapy machines in Latin American countries, such as Brazil and Venezuela, and several countries now have 1–4 MV per million population. Brachytherapy is also available in the majority of Latin American countries with other RT capacity (23, 24). The data also suggest rapid changes in available technology, which reflects the economic development and modernization in the region. From 1976 to 1999, there was a threefold increase in megavolt teletherapy machines in Southeast Asian countries (30). More recently, there has been an increase of approximately 25 machines per year in India alone (44). Although there has not been a full survey of the region’s RT resources since 2001, available data suggest that these trends are continuing in many countries. In 2004, Vietnam initiated a “National Program on Cancer Prevention.” Included in this program was a target of one oncology department per province, each one equipped with RT machines (43). There is some evidence that capacity is slowly improving with increased volume of machines and improved radiation oncology training programs, especially in Indonesia and Cambodia (31, 33). Cambodia’s University of Phnom Penh successfully partnered with international centers and universities to provide training for oncologists (31). There also have been efforts from National Cancer Institute Center of Global Health, IAEA, Union for International Cancer Control (UICC), and academic centers in the United States to help narrow the gap in RT access and training. Many of these collaborations are still developing and require persistent effort from institutions in the US and other developing countries to make these collaborations productive and successful (60–63). African organization on Research and Training in Cancer (AORTIC) has also been leading several efforts in improving cancer care capacity in Africa (64).

Comparing the numbers of RT centers and machines enumerated by the literature in the systematic review to DIRAC, we found the literature to be out of date. Of all non-DIRAC reports included in the review, only 14 unique articles provided updated numbers for a total of 11 countries. With the exception of Indonesia, Mexico, and Turkey, most were written prior to 2008 and were no longer accurate. Many of these countries may have their own national cancer registries and databases for RT resources, but they do not appear to be publishing on this data. This may suggest that established international databases, such as DIRAC, may be sufficient and comprehensive enough to serve as the primary sources for global radiation equipment inventory. National registries may then be used for other purposes such as directing resources toward regions that need machine maintenance and replacement or informing decisions on where to develop new RT resources.

The primary strength of this study is the robustness of the search strategy. The thoroughness of the search terms and wide scope of sources searched ensured that very few reports were missed. However, despite the robustness of the search, the review is mainly limited by data availability. While it is likely that the lack of information is directly correlated to a lack of RT services, it is also possible that institutions lack incentives to report on RT services given DIRAC’s international presence and historically regular reporting. Furthermore, it is important to note that treatment of cancer requires capacity in a variety of areas in addition to RT such as radiology, surgery, medical oncology, and pathology. Therefore, this review presents a small but significant aspect of the cancer care continuum. We acknowledge that delineating the challenges of radiation capacity does not capture the entire picture of access and delivery of cancer treatment.

## Conclusion

Though many LMIC struggle to meet the demand for radiation therapy delivery, few reports exist in the literature about these issues. This systematic review identifies major challenges to delivering RT in these regions, including lack of physical resources, lack of human personnel, and lack of data. DIRAC reports and online resources likely reflect real-time changes in RT capacity, but non-DIRAC-originated reports tend to be out of date, even in countries with national cancer registries. Institutions should publish more data on their capacity to deliver RT and the specific challenges they face; only then can interventions aimed at mitigating these issues be developed. Where possible, neighboring countries should collaborate and share resources to improve the scope of RT delivery, particularly when there is an economic disparity between neighboring countries. Furthermore, international funding agencies should make increasing RT capacity in LMIC a priority.

## Conflict of Interest Statement

The authors declare that the research was conducted in the absence of any commercial or financial relationships that could be construed as a potential conflict of interest.

## Supplementary Material

The Supplementary Material for this article can be found online at http://www.frontiersin.org/Journal/10.3389/fonc.2014.00380/abstract
