# Supplementary material for: A Systematic Review of Radiotherapy Capacity in Low- and Middle-Income Countries
Source: Front Oncol. 2015 Jan 22;4:380. doi: 10.3389/fonc.2014.00380 (PMC4302829; doi:10.3389/fonc.2014.00380)
Supplement: Supplementary file 1 [file DataSheet_1.PDF]

REVIEWED ARTICLES BY REGION

Africa:

1. Campbell OB, Arowojolu AO, Akinlade BI, Adenipekun A, Babarinsa IA. Advanced cervical carcinoma in Ibadan, Nigeria: an appraisal of radiation therapy. Journal of Obstetrics & Gynecology 2000;20(6):624-627.

2. Denny L, Anorlu R. Cervical cancer in Africa. Cancer Epidemiol Biomarkers Prev 2012 Sep;21(9):1434-1438.

3. Denny L. Cervical cancer treatment in Africa. Curr Opin Oncol 2011 Sep;23(5):469-474.

4. Duncan JT. Medical application of radiation in Nigeria. J Natl Med Assoc 1972 Jul;64(4):366-369.

5. Durosinmi-Etti F. An overview of cancer management by radiotherapy in Anglophone West Africa. International Journal of Radiation Oncology* Biology* Physics 1990;19(5):1263-1266.

6. Dehaeck K, Lombard J. The natural history of carcinoma of the cervix in young women. SAMJ 1992;82:351.

7. Airede L, Onakewhor J, Aziken M, Ande A, Aligbe J. Carcinoma Of The Uterine Cervix In Nigerian Women: The Need to Adopt a National Prevention Strategy. Sahel Medical Journal 2008;11(1):1-11.

8. Abdel-Wahab M, Bourque J, Pynda Y, Iżewska J, Van der Merwe D, Zubizarreta E, et al. Status of radiotherapy resources in Africa: an International Atomic Energy Agency analysis. The lancet oncology 2013;14(4):e168-e175.

9. Adewuyi SA, Ajekigbe AT, Campbell OB, Mbibu NH, Oguntayo AO, Kolawole AO, et al. Pattern of oncologic emergencies seen in adult cancer patients attending the Radiotherapy and Oncology Centre, Ahmadu Bello University Teaching Hospital, Zaria - Nigeria. Niger Postgrad Med J 2012 Dec;19(4):208-214.

10. Ikechebelu J, Onyiaorah I, Ugboaja J, Anyiam D, Eleje G. Clinicopathological analysis of cervical cancer seen in a tertiary health facility in Nnewi, south-east Nigeria. Journal of Obstetrics & Gynaecology 2010;30(3):299-301.

11. Levin CV, Sitas F, Odes RA. Radiation therapy services in South Africa. S Afr Med J 1994 Jun;84(6):349-351.

12. Martin WMC. Radiotherapy in developing countries. Br J Radiol 1993;66(782):220-224.

13. Wabinga H, Ramanakumar A, Banura C, Luwaga A, Nambooze S, Parkin D. Survival of cervix cancer patients in Kampala, Uganda: 1995–1997. Br J Cancer 2003;89(1):65-69. doi: 10.1038/sj.bjc.6601034

14. Levin CV, El Gueddari B, Meghzifene A. Radiation therapy in Africa: distribution and equipment. Radiotherapy and Oncology 1999;52(1):79-83.

15. Thomas J. Cancer control in Africa: a call for action. Afr J Med Med Sci 2004 Mar;33(1):1-4.

16. Ago BU, Agan TU, Ekanem EI. Cancer of the uterine cervix at the University of Calabar Teaching Hospital, Calabar Nigeria. Cancer Research Journal 2013;1(4):37-40.

Americas:

1. Souhami L. Quality assurance in radiation therapy: Clinical aspects. International Journal of Radiation Oncology* Biology* Physics 1984;10:69-72.

2. Poitevin-Chacón A, Hinojosa-Gómez J. Patterns of care of radiotherapy in México. Reports of Practical Oncology & Radiotherapy 2013;18(2):57-60.

3. Zubizarreta EH, Poitevin A, Levin CV. Overview of radiotherapy resources in Latin America: a survey by the International Atomic Energy Agency (IAEA). Radiotherapy and oncology 2004;73(1):97-100.

4. Calmon Teixeira L. Situation of radiotherapy in Latin America. International Journal of Radiation Oncology* Biology* Physics 1990;19(5):1267-1270.

5. Candelaria M, Cetina L, Garcia-Arias A, Lopez-Graniel C, de la Garza J, Robles E, et al. Radiation-sparing managements for cervical cancer: a developing countries perspective. World J Surg Oncol 2006 Nov 13;4:77.

Asia:

1. Lynch HT, Rahim MA. Cancer in the Third World: Bangladesh 1980. Am J Public Health 1981 Oct;71(10):1158-1161.

2. Dinshaw K. Radiation oncology: the Indian scenario. International Journal of Radiation Oncology* Biology* Physics 1996;36(4):941-943.

3. Fu LT. Radiotherapy in China today. International Journal of Radiation Oncology* Biology* Physics 1989;16(2):293-295.

4. Martin WC. Radiotherapy and Oncology in Papua New Guinea-How it Differs from Western Practice. Australas Radiol 1990;34(3):238-240.

5. Tatsuzaki H, Levin CV. Quantitative status of resources for radiation therapy in Asia and Pacific region. Radiotherapy and Oncology 2001;60(1):81-89.

6. Eav S, Schraub S, Dufour P, Taisant D, Ra C, Bunda P. Oncology in Cambodia. Oncology 2012;82(5):269-274.

7. Goksel F, Koc O, Ozgul N, Gultekin M, Abacioglu M, Tuncer M, et al. Radiation oncology facilities in Turkey: Current status and future perspectives. Asian Pac J Cancer Prev 2011;12(9):2157-2162.

8. Gondhowiardjo S, Prajogi G, Sekarutami S. History and growth of radiation oncology in Indonesia. Biomed Imaging Interv J 2008;4(3):e42.

9. Biswas LN, Deb AR, Pal S. Radiation therapy: experience in lndian patients. J Indian Med Assoc 2005 Sep;103(9):486-488.

10. Prasiko G, Jha A, Dong J, Srivastava R. Experience of Brachytherapy in in Cacinoma of uterine Cervix at BP Koirala Memorial cancer Hospital, Bharatpur, Chitwan, Nepal. Journal of Nepal Medical Association 2004;43(151):19-22.

11. Malik S, Banu PA, Rukhsana N, Ahmed M, Yasmin Z. A comprehensive study on HDR brachytherapy treatments of cervical cancers: using the first Co-60 BEBIG Multisource Unit in Bangladesh. J Contemp Brachyther 2011;3(2):96-105.

12. Mahantshetty U, Krishnatry R, Kumar S, Engineer R, Maheshwari A, Kerkar R, et al. Consensus meeting and update on existing guidelines for management of cervical cancer with special emphasis on the practice in developing countries, including India: The expert panel at the 8th annual women's cancer initiative Tata Memorial Hospital Conference 2010-11. Indian journal of medical and paediatric oncology 2012;33(4):216. doi: 10.4103/0971-5851.107083

13. Lertbutsayanukul C, Lertsanguansinchai P, Shotelersuk K, Khorprasert C, Rojpornpradit P, Asavametha N, et al. Results of radiation therapy in stage 1B cervical carcinoma at King Chulalongkorn Memorial Hospital: fifteen-year experience. J Med Assoc Thai 2001 Jun;84 Suppl 1:S216-27.

14. Liu S, Huang X, Ke G, Huang X. 3D radiation therapy or intensity-modulated radiotherapy for recurrent and metastatic cervical cancer: the Shanghai Cancer Hospital experience. PloS one 2012;7(6):e40299.

15. Domingo EJ, Dy Echo, Ana Victoria V. Epidemiology, prevention and treatment of cervical cancer in the Philippines. Journal of gynecologic oncology 2009;20(1):11-16.

16. Manusirivithaya S, Sripramote M, Tangjitgamol S, Sanjareonsuttikul N, Pisarnturakit P. Cost effectiveness of concurrent chemoradiation in comparison with radiation alone in locally advanced cervical cancer. J Med Assoc Thai 2005 Aug;88(8):1035-1044.

17. Ma J, Zhu Q, Han S, Zhang Y, Ou W, Wang H, et al. Effect of socio-economic factors on delayed access to health care among Chinese cervical cancer patients with late rectal complications after radiotherapy. Gynecol Oncol 2012;124(3):395-398.

18. To DA, Bui D. Current status of radiotherapy in Vietnam, 2002. Radiat Med 2004 Jan-Feb;22(1):12-16.

19. Ravichandran R. Has the time come for doing away with Cobalt-60 teletherapy for cancer treatments. J Med Phys 2009 Apr;34(2):63-65

20. Chandel SS, Singh KK, Nigam AK, Baghel RS. "The effect of treatment prolongation in treatment of cervical cancer patient" – treated patients at rural center in India. IOSR-JDMS 2013;9(2):70-75.

Europe:

1. Guedea F, Venselaar J, Hoskin P, Hellebust TP, Peiffert D, Londres B, et al. Patterns of care for brachytherapy in Europe: updated results. Radiother Oncol 2010 Dec;97(3):514-520.

2. Rosenblatt E, Izewska J, Anacak Y, Pynda Y, Scalliet P, Boniol M, et al. Radiotherapy capacity in European countries: an analysis of the Directory of Radiotherapy Centres (DIRAC) database. Lancet Oncol 2013 Feb;14(2):e79-86.

Multiple Countries:

1. Levin V, Tatsuzaki H. Radiotherapy services in countries in transition: gross national income per capita as a significant factor. Radiotherapy and oncology 2002;63(2):147-150.

2. Hanson G, Stjernswärd J, Nofal M, Durosinmi-Etti F. An overview of the situation in radiotherapy with emphasis on the developing countries. International Journal of Radiation Oncology* Biology* Physics 1990;19(5):1257-1261.

3. Barton MB, Frommer M, Shafiq J. Role of radiotherapy in cancer control in low-income and middle-income countries. The lancet oncology 2006;7(7):584-595.

4. Zaidi H. Medical physics in developing countries: looking for a better world. Biomed Imaging Interv J 2008;4:1-5.

5. Ansink AC. Cervical cancer in developing countries: how can we reduce the burden? Awareness raising, screening, treatment and palliation. Trop Doct 2007 Apr;37(2):67-70.

6. Kitchener HC, Hoskins W, Small W,Jr, Thomas GM, Trimble EL, Cervical Cancer Consensus Group. The development of priority cervical cancer trials: a Gynecologic Cancer InterGroup report. Int J Gynecol Cancer 2010 Aug;20(6):1092-1100.

7. Martin W. Cancer in developing countries: Part I—cancer burden, resources, epidemiology, aetiology and clinical practice. Clin Oncol 1998;10(4):219-225.
